# Interventional or surgical management of iatrogenic ostial coronary dissections

**DOI:** 10.1186/1749-8090-8-S1-O167

**Published:** 2013-09-11

**Authors:** R Babic, M Farkic, S Borovic

**Affiliations:** 1 Dedinje Cardiovascular Institute, Belgrade, Serbia; 2 Belgrade University School of Medicine, Belgrade, Serbia

## Background

Iatrogenic ostial coronary dissections (IOCD) are rare, albeit seemingly underreported complications of coronary diagnostic and interventional procedures. Its imminent ischemic and hemodynamic consequences urge prompt reaction: interventional or surgical procedure to restore coronary flow.

## Methods

Retrospective analysis of adverse effects database at our institute over 5 years.

## Results

6 cases happened at our institution (4 on interventional and 2 on diagnostic procedures) and 1 was urgently transferred from a secondary center without on site surgery following coronary intervention. Of 7 cases, 2 happened with radial approach and 5 with femoral. Ostial LM was dissected in 4 cases and RCA in 3. Aortic propagation less than 2.5 cm was seen in 2 pts. Five patients underwent interventional revascularization and 2 surgical (1 was stable (RCA) and 1 unstable (LM)). All 5 interventions were successful with prolonged hospital stay and uneventful f-up. Surgical intervention on RCA dissection was successful with uneventful f-up, patient with LM dissection underwent rescue surgery with double SV grafting, but died however at 4^th^ postoperative day of intractable heart failure due to large myocardial infarction. Of 4 LM dissections, 2 were treated with 1 ostial stent, one with 2 sequential stents, and one with 2 stents in LM (culotte stenting). Two RCAs was stented with 1 stent, and one needed 3 stents to seal and prevent further aorto-ostial propagation.

## Conclusions

Interventional treatment of IOCD dissection is feasible requiring skills and experienced team work. Surgical treatment is good alternative if delivered immediately and if basic hemodynamics could be maintained up to the operation theater.

